# Complementary and alternative medicine use in migraine patients: results from a national patient e-survey

**DOI:** 10.3389/fneur.2024.1378532

**Published:** 2024-05-28

**Authors:** Gytis Makarevičius, Austėja Dapkutė, Kristina Ryliškienė

**Affiliations:** Center of Neurology, Faculty of Medicine, Vilnius University, Vilnius, Lithuania

**Keywords:** complementary, alternative, migraine, cannabis, personality traits, health worries

## Abstract

**Object:**

This cross-sectional study aims to investigate migraineurs’ preferred complementary and alternative medicine (CAM) types and the factors influencing their usage.

**Materials and methods:**

An anonymous e-survey was distributed to Lithuanian Migraine Association members, and social media migraine support communities. The collected data consisted of demographic, migraine-related questions, personal qualities, CAM habits.

**Results:**

470 respondents were analyzed. 95.96% were women with a median age of 37 (IQR 31, 44). The median duration of migraine was 17.5 years (IQR 10, 25) and the median headache severity was rated 8 (IQR 7, 10) out of 10. 68.90% of participants had one or more headache days per week. 71.49% of respondents were triptan users, 27.66% used medical prophylaxis, and 17.87% used monoclonal antibodies. 52.55% of respondents used CAM in the past 12 months. Physical activity (36.17%), dietary changes/fasting (27.02%), relaxation/meditation (26.60%) were the most used CAM types. Reasons for CAM use included dissatisfaction with conventional treatment effectiveness (42.51%), concerns about safety (48.18%) and adverse effects (37.25%). Factors associated with the decision to explore CAM included longer headache duration (*p* = 0.017, Mann–Whitney U test), frequent sick leaves (*p* < 0.001, Mann–Whitney U test), current preventive medication use (*p* = 0.016, chi-square test), positive views on CAM safety/naturality (*p* = 0.001/ *p* < 0.001, Mann–Whitney U test), belief of having a healthy diet (p < 0.001, chi-square test), food-related worries (*p* = 0.011, Mann–Whitney U test) and Big-five personality trait of openness to experience (*p* = 0.049, chi-square test). After logistic regression, the frequent need to take sick leaves, having a healthy diet, food-associated fears maintained statistical significance. CAM use was not associated with non-adherence to conventional medicine. 48.99% of CAM consumers disclosed CAM use to their doctors.

**Conclusion:**

CAM is explored by a significant proportion of migraineurs, less than half communicate this to their doctors. In our sample, physical activity, dietary changes, and relaxation techniques were the most common. Many patients opted for CAM due to previously experienced side effects/ineffectiveness of conventional migraine treatment or the fear of potential harm from standard medication. Individual factors, such as openness of personality can be an important contributing factor.

## 1 Introduction

Migraine is a neurological disorder characterized by severe headache attacks greatly affecting patients’ quality of life and work performance (1). Complementary and alternative medicine (CAM) is defined as a group of diverse medical and health care systems, practices, and products that are not generally considered part of conventional medicine (2). Migraine patients usually require long-term treatment due to frequent attacks or a chronic course of the disorder (1). As in other chronic illnesses (2–4), migraine sufferers often turn to CAM, either alongside or instead of conventional medical treatment. Based on published data so far, over 40% of adults with migraine use complementary and alternative medicine (5–7). Although CAM for migraine is widely used in real life, there is limited research on its effectiveness and safety, as well as patients’ perceptions of these treatments (5). Only a fraction of CAM methods used in migraine have scientific evidence and are recommended by the German Migraine and Headache Society and the German Society of Neurology (those include acupuncture, biofeedback and aerobic-physical exercise). Others, including homeopathy, reflexological massage, piercings and elimination diet, lack evidence and are typically not recommended (8). Thus, it is imperative for medical specialists to understand patients’ perspectives on CAM for migraines to better guide their treatment. Without proper management, CAM might also pose unforeseen dangers. For example, butterbur, that is used as an alternative treatment for migraine prevention, can predispose liver toxicity, allergic reactions (9). Cannabis use could result in substance abuse related problems, exacerbation of psychotic disorders, mood disorders, neurocognitive impairments (10). Fasting and diets, could result in nutritional imbalances (11, 12) and strenuous unmonitored physical exercise, especially, for former sedentary individuals with cardiovascular diseases, can cause acute cardiac events (13). Despite this, in comparison to conventional medical treatment, CAM is often perceived by society as safer and more “natural” (14). Such thinking might lead more concerned patients to substitute standard migraine care, potentially resulting in a higher rate of treatment failure.

This cross-sectional study aims to identify CAM prevalence among migraineurs in Lithuania, reasons, migraine related and personality factors influencing its utilization, and examine its impact on patient compliance with conventional physician-prescribed treatments.

## 2 Materials and methods

### 2.1 Questionnaire development

Initially, we conducted a literature review in online databases (PubMed: National Library of Medicine, Google Scholar) to identify factors associated with CAM use. Search-keywords included (“alternative medicine” OR “complementary medicine”) AND (“corelate” OR “factor” OR “predictor” OR “trait”). Search-criteria included: full text articles, systematic reviews and/or meta-analyses, written in English between 2016 and 2021, discussing CAM use and possible associated factors in general populations. Articles discussing CAM use in pediatric populations, also those describing CAM use in people with professional medical knowledge (health care professionals, trainees or students) were excluded. 6 articles remained for further review (14–19). Galbraith et al. extensively reviewed personality traits and cognitions as factors associated with CAM use, while Tangkiatkumjai et al. highlighted perceived safety and naturality, dissatisfaction with conventional treatment (14, 19). These findings prompted us to test the influence of Big-five traits, opinions regarding CAM safety, naturality, perceived control over one’s health, modern health worries between migraineurs.

For the purpose of our study, we defined and specified to our respondents that CAM should be viewed as all possible practices, devices, products and actions a person could use thinking that it will alleviate migraine attack and/or reduce the frequency of migraine attacks that are not generally considered part of conventional medicine (i.e., physical activity, homeopathy, herbs, relaxation techniques, vitamins/ minerals, diets, fasting, etc.). Conventional medicine options (including neuromodulation devices) that were prescribed by a doctor are not considered CAM in this study. The non-invasive nerve stimulation devices were not considered “complementary and/or alternative” as these treatments have been approved by the United States Food and Drug Administration and have clear indications for their use (8, 20). Information about CAM usage in the last 12 months was collected. CAM was classified into 8 groups (Table 1). For each of the different group respondents received individual questions with a list of the most common examples to choose from Table 1 and an option to provide alternative response. For oral substances, an open question asking to name used substances was given. Any orally administrated substance was accepted, however, the results were reviewed and Magnesium, Coenzyme q10 and Riboflavin (Vitamin B_2_) were disregarded. Similarly, inputs more fitting to other CAM classes, for example “drinking cannabis oil,” were classified to appropriate group instead. Any substance applied to the skin or on the skin was considered a Topical treatment (Table 1). Regarding physical activity, any movement which was used for migraine attack and/or prevention were included. Yoga was also classified to physical activities instead of relaxation techniques or meditation group, although we acknowledge the duality of this practice. Massage, osteopathy and chiropractic were defined as follows (Table 1) and was not further explained. Respondents could choose whether CAM they used qualifies to this group, no possibility to give alternative answer to this group was provided. “Other” option was not provided for Acupuncture/Acupressure group, as well. Additionally, a multiple-choice question regarding self-reported reasons for using CAM and a question whether respondent reported CAM to the doctor were included.

**Table 1:** Examples of each complementary and alternative medicine group.

CAM category	Examples
Oral substances	Phytotherapy, herbs, homeopathy, traditional Chinese medicine, natural substances, vitamins/minerals^*^
Topical treatments	Cold/heat applications, essential oils, herbal compress, ointments
Acupuncture/Acupressure	Acupuncture and/or acupressure
Relaxation techniques or meditation	Relaxation/ breathing exercises, meditation, hydrotherapy, aromatherapy, music therapy, visualization, mindfulness, chromotherapy, art therapy
Fasting, “trigger” elimination or any other diet	Fasting, intermittent fasting, low-carbohydrate diet, low-sugar diet, ketogenic diet, lactose-free diet, vegetarian diet, veganism, mediterranean diet, gluten-free diet, “trigger” avoidance
Cannabis and its oils^**^	Cannabis smoking, cannabis tea, cannabidiol oil, other cannabidiol supplements (capsules, gummies, etc.)
Massage, osteopathy and chiropractic	Massage, osteopathy and/or chiropractic
Physical activity	Walking, jogging, dancing, swimming, cycling, yoga^***^, stretching exercises, aerobics

^*^ Users of Magnesium, Coenzyme q10 and Riboflavin (Vitamin B_2_) were excluded. ^**^Cannabis tea, Cannabidiol oil/other substances users were sorted to Cannabis and its oils and not to Oral substances or Topical treatments group. ^***^Yoga was sorted to physical activity group and not relaxation techniques or meditation group in our study.

Personality traits were assessed with a Lithuanian version of Big-five traits questionnaire (21). The questionnaire consists of 25 pairs of opposing adjectives. For each pair, the respondent was asked to choose one that better applies to themselves in a scale from 1 to 7 (the higher the number the more fitting is the opposing adjective; Supplementary material). The scale consists of 5 parts: neuroticism, extraversion, openness, agreeableness and conscientiousness. The Cronbach’s alpha for each part in our study were: 0.70 for neuroticism, 0.53 for extraversion, 0.51 for openness, 0.70 for agreeableness and 0.60 for conscientiousness. To measure other psychological factors, we created individual five-point Likert-scale-type questions ranging from “strongly agree” to “strongly disagree” for statements “CAM is natural,” “CAM is safer than conventional medical treatment,” “My health mostly depends on my actions.” A total of 5 different questions regarding modern health worries were composed one for 5 different areas: food, drinking water, radiation, medical diagnostics and vaccines. Each of the areas were detailed to the respondents. For example, food associated worries—concerns that pesticides, bacteria, additives, microplastics in food could negatively affect one’s health (Supplementary material). The answers were evaluated by five-point Likert scale from “no worries” to “strong worries.”

General and demographic data included age, sex, education, household situation, residence, use of nicotine products, general health perception, comorbidities and yes/no questions regarding personal perceptions of maintaining a healthy diet and sufficient physical activity.

Migraine characteristics included migraine type, age of migraine onset, average headache severity in numerical rating scale (from 1 to 10) when acute medication was not effective/not used, average frequency and duration of an attack (frequency scale, Table 2), impact on work ability (four-point Likert-type scale, Table 2), the need to take sick leave due to an attack (four-point Likert-type scale, Table 2), medication overuse (single choice question – yes/no/do not know).

**Table 2:** Demographic and migraine characteristics of survey respondents (*n* = 470).

	Median (Q1, Q3); (MIN, MAX)	Frequency (%)
**Age (years)**		
Sex	37 (31, 44); (14, 68)	
Female		451 (95.96)
**Education**		
Higher		382 (81.28)
Vocational		42 (8.94)
High school		43 (9.15)
Less than high school		3 (1.28)
**Family status**		
Living alone		74 (15.74)
Single parent		26 (5.53)
Living with partner without children		112 (23.83)
Living with partner and children		247 (52.55)
Other		11 (2.34)
**Residence**		
District center		287 (61.06)
Other		183 (38.94)
**Comorbidities**		
Anxiety		120 (25.53)
Depression		35 (7.45)
Sleep disorders		80 (17.02)
Gastrointestinal disorders		124 (26.38)
Endocrine disorders		73 (15.53)
Gynecological disorders		74 (15.74)
Cardiovascular disorders		85 (18.09)
Other neurological disorders		10 (2.13)
None		124 (26.38)
Years lived with migraine	17.5 (10, 25); (0, 54)	
**Migraine type**		
Migraine with aura		162 (34.47)
Average headache severity (in NPRS)	8 (7, 10); (1, 10)	
**Average headache duration**		
Up to 1 h		9 (1.91)
1–4 h		59 (12.55)
4–12 h		124 (26.38)
12–24 h		103 (21.91)
24 and more hours		175 (37.24)
**Headache frequency**		
Less than 1 day a month		50 (10.64)
1 day a month—less than 1 day in a week		96 (20.43)
1 day a week		118 (25.11)
2–3 days in a week		133 (28.30)
4–5 days in a week		32 (6.81)
Almost every day/ every day		41 (8.72)
**Does migraine attack impact your ability to work**		
Never		4 (0.85)
Rarely		75 (15.96)
Often		254 (54.04)
Always		137 (29.15)
**Need to take sick-leave during migraine attack**		
Never		234 (49.79)
Rarely		143 (30.43)
Often		67 (14.26)
Always		26 (5.11)
Use of abortive medication		442 (94.04)
Triptan use		336 (71.49)
**Effectiveness** ^*^ **of abortive medication**		
Never		5 (1.13)
Rarely		69 (15.61)
Often		337 (76.24)
Always		31 (7.01)
Use of preventive medication		130 (27.66)
Use of anti-CGRP/Rc mAb		84 (17.87)
Medication-overuse		65 (13.83)
Always adheres to doctors’ recommendations		256 (57.14)

NPRS, The Numeric Pain Rating Scale. Anti-CGRP/Rc mAb, monoclonal antibodies against calcitonin gene-related peptide or receptor. ^*^Abortive medication was considered effective if headache subsided within 2 h.

Conventional migraine treatment part included multiple-choice acute and preventive medication list and five-point Likert-type question ranging from “always” to “never” regarding self-reported adherence to prescriptions. Respondents who reported not always adhering to doctor’s recommendations were regarded as non-adherent. Effectiveness of abortive medication in four-point Likert-type scale (Table 2) was measured.

An initial questionnaire was sent to 5 active members of the Lithuanian Migraine Association (Patient Association). Based on their feedback, minimal adjustments were made for formatting and accuracy. Finally, the pilot questionnaire was tested on the same patient group for functionality.

### 2.2 Data collection

The survey was administered using Microsoft forms and distributed via email to members of the Lithuanian Migraine Association, as well as shared on migraine support groups on Facebook. The survey remained open from 15 December 2021 to 15 January 2022. A reminder was sent 2 weeks after the initial email. The survey was anonymous and voluntary. Only data of respondents with a self-reported confirmed migraine diagnosis were included in the final analysis. Ethics approval for this study was not required according to the Vilnius Regional Biomedical Research Ethics Committee, as the received data were non-identifiable (General Data Protection Regulation Principle 26). Neither the survey respondents nor the Lithuanian Migraine Association received any financial compensation.

### 2.3 Statistical analysis

Descriptive statistics were employed to summarize demographic, migraine characteristics of respondents and the purposes of CAM use. Dichotomous and nominal data were presented as raw numbers and percentages of the total. Numerical data were summarized using the median and interquartile range (given the absence of normally distributed numerical data in the dataset). The Lilliefors test was used to test for normality and the two-variances F-test for homogeneity of data. The Mann–Whitney U test was utilized to compare differences in ordinal and interval data between CAM user and non-user groups. For dichotomous variables, chi-square analysis was employed to compare differences between the groups. To identify factors associated with CAM use, a backward elimination stepwise variable selection was conducted. The selected variables were then included in a final model of binomial logistic regression. All statistical analyses were performed using Microsoft 365 Excel and R software (version 4.1.2). A generalized linear model (*GLM*) function was applied to develop a full binary logistic regression model. The Akaike Information Criteria (*StepAIC*) function from the MASS package was used to perform backward variable selection. The selected variables were subsequently included in a final binary logistic regression model. A significance level of *p* < 0.05 was chosen. The multivariable logistic regression was chosen due to its ability to simultaneously examine multiple predictors. We also chose multivariable logistic regression as it can account for the complex interplay among multiple predictors and provide more nuanced insights into the strength and direction of associations with a binary outcome variable. Given the complex nature of human behavior and decision-making we did not strive to produce a usable predictive model.

## 3 Results

### 3.1 Study population

A total of 470 out of 555 respondents with a self-reported confirmed migraine diagnosis were included in the final analysis. The summary of demographic and migraine characteristics is presented in Table 2.

### 3.2 Complementary and alternative medicine use

**Figure 1:**
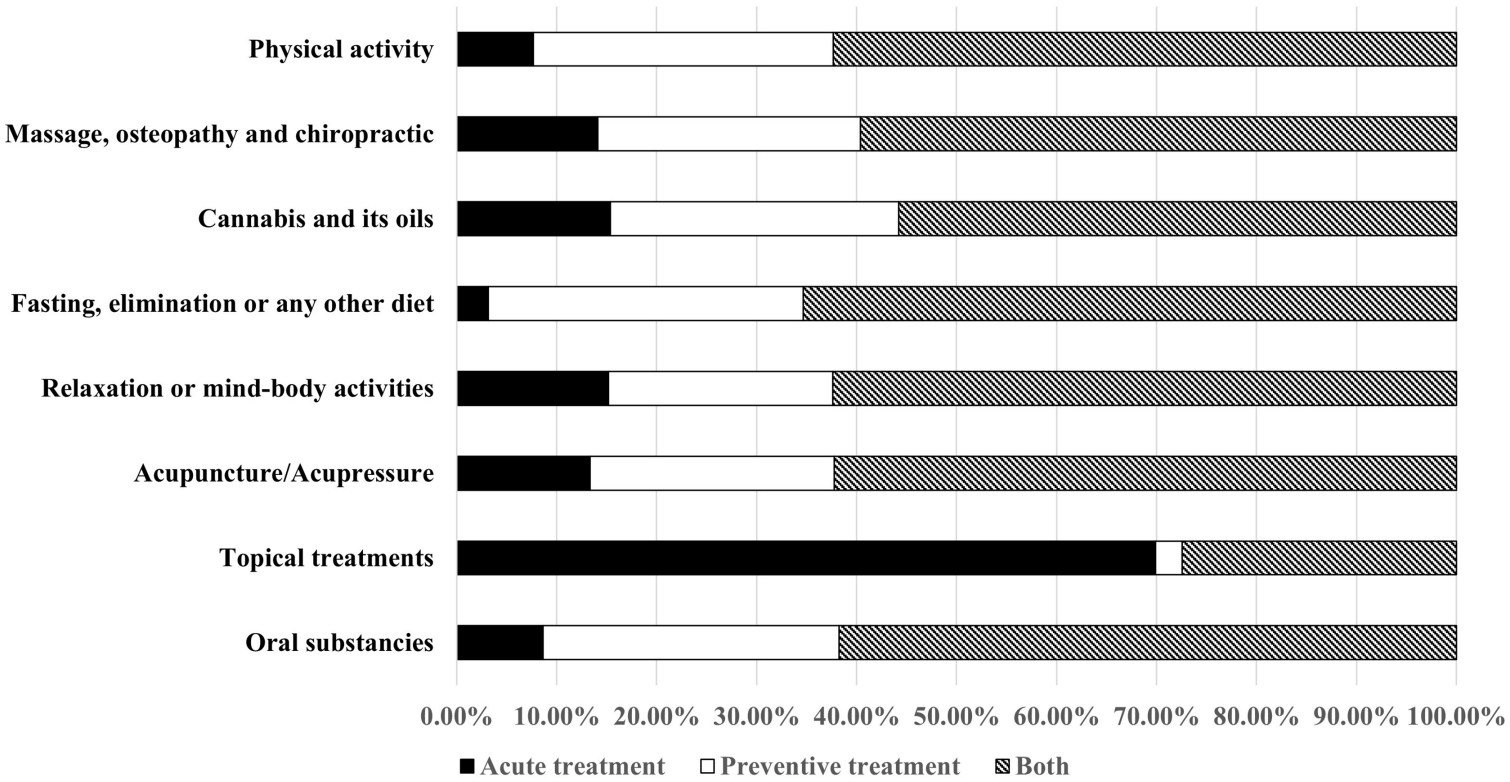
Complementary and alternative medicine use for acute treatment, and/or migraine prophylaxis.

247 (52.55%) respondents reported the use of complementary and alternative medicine (CAM) for migraine in the past 12 months. The 3 most utilized CAM groups among the 8 suggested were physical activity (walking 27.65%; yoga/stretching exercises 15.11%; swimming 5.96%), dietary changes and fasting (low sugar/ low carbohydrate diet 13.83%; lactose-free diet 8.51%; “trigger” product elimination 8.09%; fasting 3.83%), relaxation and meditation (relaxation or breathing exercises 16.60%; meditation 15.32%; hydrotherapy 8.94%). Other types, including massage, osteopathy, and chiropractic (21.06%), acupuncture/acupressure (9.57%), topical treatments (24.04%), cannabis (11.06%) and oral treatments (15.53%) were used less commonly. The median number of different CAM groups used by one respondent was 3 (IQR 2, 4). The most common CAM combinations were physical activity plus relaxation/meditation (40.89%), physical activity plus dietary changes/fasting (38.87%), and relaxation/meditation plus dietary changes/fasting (31.17%). A triple combination of physical activity plus relaxation/meditation plus dietary changes/fasting was used by 27.13% of CAM users. All CAM groups, except for topical ailments, were more commonly used as a preventive measure rather than acute treatment for an ongoing attack (Figure 1). Notably, 121 out of 247 (48.99%) CAM users in the past 12 months disclosed this use to their doctors.

### 3.3 Factors associated with CAM use

**Figure 2:**
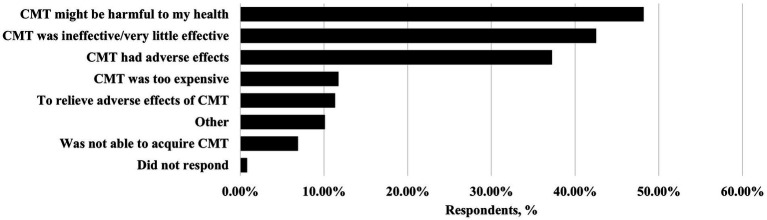
Reasons for using complementary and alternative medicine in migraine patients. CMT, conventional migraine treatment. Percentages add up to more than 100 as a person could have chosen more than one reason.

Reported reasons for using complementary and alternative medicine are depicted in Figure 2. The use of CAM was found to be associated with a longer headache duration (*p = 0.017,* Mann–Whitney U test), more frequent sick leaves (*p < 0.001,* Mann–Whitney U test) and the usage of preventive medication (*p = 0.016,* chi-square test). CAM users also reported more positive opinions on CAM than non-users in terms of safety (*p = 0.001,* Mann–Whitney U test) and naturality (*p < 0.001,* Mann–Whitney U test). They also had more concerns about food safety (*p = 0.011*, Mann–Whitney U test) and were more likely to report having a healthy diet (64.8% vs. 44.4%, *p < 0.001*; chi-square test). The predominant personality trait of openness was associated with CAM use (9.7% vs. 4.9%, *p = 0.049*; chi-square test), while neuroticism was more common in the non-user group (9.4% vs. 3.2%, *p = 0.005;* chi-square test). CAM use was not associated with non-adherence (*p = 0.399;* chi-square test).

Oral CAM treatment users expressed more pronounced modern health worries with higher concerns about food, drinking water, and radiation-associated worries (*p = 0.007, p = 0.001, p = 0.035;* Mann–Whitney U test). Respondents using relaxation techniques, cannabis or massage/osteopathy were more likely to have openness to experience as their predominant personality trait (12.0% vs. 5.80%, *p = 0.024;* 15.38% vs. 6.46%, *p = 0.021;* 12.12% vs. 6.20%, *p = 0.046*, respectively; chi-square test). Patients using relaxation techniques were more positive about the statement that one’s health mostly depends on one’s actions (*p = 0.003,* Mann–Whitney U test). Cannabis users were younger (33.5 vs. 37.0, *p = 0.016,* Mann–Whitney U test), whereas those pursuing diet changes were older (39.0 vs. 37.0, *p = 0.006,* Mann–Whitney U test), with a higher percentage living in rural areas (47.24% vs. 35.86%, *p = 0.025,* chi-square test).

After a backward elimination stepwise variable selection and subsequent logistic regression, only having a healthy diet, the need to take sick leave for migraine attacks and food-associated worries maintained consistent statistical significance, contributing to a higher probability of CAM use (Table 3).

**Table 3:** Factors included in multivariable logistic regression model for complementary and alternative medicine use.

Factor	Estimate	SE	OR (95% CI)	*p*-value
Intercept	−0.451	0.791	0.637 (0.133, 3.015)	0.569
Frequency	0.134	0.077	1.144 (0.985, 1.331)	0.079
**Use of abortive medication**				
NO (ref)	-	-	-	-
YES	−0.864	0.457	0.421 (0.165, 1.005)	0.059
**Need to take sick leave during migraine attack**				
Never (ref)	-	-	-	-
Rarely	0.662	0.24	1.938 (1.214, 3.115)	**0.005**
Often	0.807	0.311	2.242 (1.227, 4.170)	**0.014**
Always	0.728	0.484	2.071 (0.817, 5.533)	0.163
**Do you believe you have a healthy diet?**				
NO (ref)	-	-	-	-
YES	0.887	0.211	2.427 (1.606, 3.698)	**<0.001**
**CAM are natural**				
Strongly agree (ref)	-	-	-	-
Agree	0.321	0.582	1.379 (0.445, 4.463)	0.581
Not sure	−0.729	0.574	0.482 (0.158, 1.538)	0.204
Disagree	0.79	0.557	2.204 (0.749, 6.833)	0.156
Strongly disagree	−0.438	0.587	1.550 (0.494, 5.086)	0.457
**Drinking water associated worries**				
No worries (ref)	-	-	-	-
Minimal worries	−1.087	0.325	0.337 (0.176, 0.631)	**<0.001**
Not sure	−0.255	0.364	0.775 (0.377, 1.581)	0.483
Medium worries	−0.911	0.39	0.402 (0.185, 0.857)	**0.019**
Strong worries	−1.173	0.675	0.309 (0.082, 1.180)	0.082
**Food associated worries**				
No worries (ref)	-	-	-	-
Minimal worries	0.262	0.347	1.300 (0.661, 2.583)	0.45
Not sure	−0.18	0.533	0.835 (0.290, 2.371)	0.736
Medium worries	0.917	0.396	2.502 (1.161, 5.495)	**0.02**
Strong worries	1.1	0.501	3.004 (1.136, 8.139)	**0.028**

## 4 Discussion

Our study revealed that in the past year, a half of migraine sufferers in our sample used CAM. Respondents predominantly chose physical activity, dietary changes, and relaxation techniques. Although cannabis was not a popular option in our study, it is worth noting that non-legal status of some cannabis substances (those with tetrahydrocannabinol content more than 0.20%) in Lithuania could impact the data, potentially resulting in underreporting. Among the three most common patient-reported reasons for CAM usage, ineffectiveness of conventional medicines was reported. However, the primary reason for choosing CAM was a fear that conventional medicines might be harmful. CAM users also perceived CAM as more natural and safer compared to conventional migraine treatment and had more concerns about food safety compared to non-users. In addition, respondents with more severe symptoms, using preventive medication and requiring frequent sick leaves, were more likely to use CAM. Interestingly, we found that CAM use was more frequent among patients with more severe symptoms, as well as among preventive medicine users. This could be explained by limitations of our survey—as data on CAM usage spanned a 12-month period (“Have you used CAM during the last 12 months?”), while information on preventive medication was collected at the time of the survey (“Do you use any preventive medications?”). Consequently, accurately evaluating the relationship between CAM and preventive medication is not feasible. For example, some respondents might have used CAM alongside preventive medication due to perceived harm or dissatisfaction with conventional medicines. Others may have transitioned to preventive medication after finding CAM ineffective. In our study, the predominant personality trait of openness was associated with CAM use, especially for those choosing relaxation techniques or meditation, cannabis and massage/osteopathy/chiropractic. Respondents who take personal responsibility for their health were more likely to choose relaxation techniques or meditation.

Very similar prevalence findings to ours were previously recorded in the United States of America, where CAM was used by approximately half of migraine or severe headache sufferers (5). A systematic review of CAM utilization in neurological disorders indicated an average use of 61.6% in headaches, however, this number highly varied between studies (from 32% to 84.75%) (22). Our respondents predominantly chose physical activity, dietary changes, and relaxation techniques as preferred CAM types. This is in line with common recommendations for migraine prevention, advocating for relaxation methods, biofeedback and regular aerobic activity (8). Other CAM types, such as Mediterranean, low sugar or low carbohydrate diets, were also reported to be beneficial for general well-being and disease prevention (23, 24). Our results contradict the findings of a previous systematic review reporting traditional Chinese medicine, thermotherapy, herbal medicine and massage to be the most commonly used CAM groups by headache sufferers (22). However, it is noteworthy that this review included 6 studies with mixed headache types from different world regions (Kuwait, Turkey, USA, and United Kingdom), which could explain the data discrepancy. Nevertheless, there is still a fraction of our respondents using low-scientific-evidence CAMs, such as fasting, ketogenic diet, cannabis, oral therapies, reflexological massage and others. Beyond lacking evidence, some reports suggest these methods may even have a reverse, migraine triggering effects (8, 25–28).

A recent systemic review on neurological disorders, including multiple sclerosis, headache/migraine, stroke and epilepsy, have identified the main reasons for using CAM. These encompass a hope for symptom relief, a desire to “try everything that is possible “and a dissatisfaction with current medical treatments in terms of their effectiveness and side effects (22). Notably, this review underscores a general inclination toward CAM over conventional treatments (22). Similarly, in our study respondents pointed ineffectiveness of conventional medicines as one of the reasons for using CAM. In addition, respondents with more severe symptoms, requiring frequent sick-leaves were more likely to use CAM which could imply the need to “strengthen” ineffective treatments.

Our data also suggests that CAM use might be associated not only with the actual ineffectiveness and side effects of conventional medicines but also with opinions regarding their potential harm – the primary motivation for selecting CAM was the fear of potential harm from conventional treatments, in addition, CAM users perceived CAM as more natural and safer than conventional medicine. One of the possible explanations for this could be a belief that conventional medication is “more chemical,” unnatural, and thus, more harmful. It could also suggest that there is a lack of education on standard medicine provided to patients. An interesting finding was also that only 48.99% of CAM users reported this use to their doctors. This could point to poor doctor-patient relationships. A common reason for such non-disclosure might be a fear of a negative reaction or simply due to the doctor’s indifference toward CAM—a phenomenon which was previously reported (29). It is important to emphasize that the lack of communication could result in CAM users switching from conventional medicine to CAM, as conventional medicine is associated with more harm, side effects and ineffectiveness, while CAM is thought to be safe and natural. Unfortunately, research on this topic is limited. Some findings from epilepsy studies suggest that the use of CAM could be associated with medication non-adherence (30, 31). To note, there was no association in our study of CAM use with non-compliance with standard medicine. For optimal migraine management, healthcare providers should encourage evidence-based CAM approaches but remain cautious about possible harms (8). Finally, considering the somewhat limited training on CAM for headaches (32), improvements in medical education are also necessary. There is also a need for more extensive studies regarding the impact of CAM use on patient adherence to conventional physician-prescribed treatments, the implementation of educational programs focusing on providing evidence-based information about CAM modalities for migraine, promoting open communication between patients and providers regarding CAM use, and emphasizing the importance of integrating CAM into a comprehensive migraine management plan, barriers to discuss CAM with healthcare providers.

To our knowledge, it is the first study analyzing CAM use for migraine and its possible connection with psychological traits, personal views and disease-specific characteristics. However, this study has some limitations. Firstly, the generalizability of our results is limited. Our chosen means of online survey distribution could have contributed to lack of sampling, sampling bias (selecting for younger, better educated, digitally literate, more social respondents, respondents with access to the Internet and thus potentially people with greater access to CAM information), non-population distribution of the group with female predominance which could be in part explained by gender response tendencies to online surveys (33, 34). In fact, greater female participation is even seen in modern migraine randomized controlled trials (35). Secondly, migraine characteristic could be better evaluated by acquiring number of days with headache, number of days with migraine, special questionnaires would more thoroughly reveal migraine burden (for example The *MIDAS* (Migraine Disability Assessment) questionnaire). Thirdly, some of the compared factors divided into smaller groups resulted in relatively small sample sizes, which could have lowered the statistical power to detect differences between groups. We also acknowledge that relying on self-reported migraine diagnosis, is a limitation of our study, a questionnaire designed according to International Classification of Headache Disorders criteria would better distinguish patients with migraine diagnosis. Lastly, we assessed treatment adherence with self-reported single-choice question, which could have overestimated adherence.

## 5 Conclusion

Our study reveals a high prevalence of CAM use among migraine patients. The most utilized CAM groups include physical activity, dietary changes and fasting, relaxation, and meditation. A significant number of patients turn to CAM due to dissatisfaction with conventional treatment effectiveness, concerns of adverse effects, or fears of its potential harm. The decision to try CAM depends on multiple factors, including migraine severity and if effect on personal wellbeing, psychological factors and personal views. Alarmingly, only less than a half of CAM users admitted this use to their doctors, which might result from anticipated negative reactions. Therefore, doctors need to improve patient education regarding conventional medicines, address emerging concerns and foster open discussions about alternative medicine, thus ensuring a holistic approach to migraine care.

## Data availability statement

The original contributions presented in the study are included in the article/Supplementary material, further inquiries can be directed to the corresponding author.

## Ethics statement

Ethics approval for this study was not required in accordance with the Vilnius Regional Biomedical Research Ethics Committee, as the received data were non-identifiable (General Data Protection Regulation Principle 26). The studies were conducted in accordance with the local legislation and institutional requirements. Written informed consent for participation was not required from the participants or the participants’ legal guardians/next of kin in accordance with the national legislation and institutional requirements because the survey was anonymous and entirely voluntary.

## Author contributions

GM: Data curation, Formal analysis, Methodology, Visualization, Writing – original draft, Writing – review & editing. AD: Writing – review & editing. KR: Conceptualization, Data curation, Methodology, Supervision, Visualization, Writing – review & editing.
