# Supplementary material for: Complementary and alternative medicine use in migraine patients: results from a national patient e-survey
Source: Front Neurol. 2024 May 28;15:1378532. doi: 10.3389/fneur.2024.1378532 (PMC11165229; doi:10.3389/fneur.2024.1378532)
Supplement: Supplementary file 2 [file Data_Sheet_1.PDF]

ALTERNATYVIOS MEDICINOS PRIEMONIŲ NAUDOJIMAS: MIGRENA SERGANČIŲJŲ APKLAUSA

1. **Patvirtinu, kad sutikimą dalyvauti šiame tyrime duodu laisva valia ir sutinku, kad apibendrinti mano anoniminiai atsakymai būtų naudojami mokslinėje veikloje.**

○ Sutinku

○ Nesutinku

1. BENDROJI INFORMACIJA IR BENDRA SVEIKATOS BŪKLĖ

2. **Koks Jūsų amžius?**

_________ *m.*

3. **Kokia Jūsų lytis?**

○ Moteris

○ Vyras

4. **Koks Jūsų išsilavinimas**

○ Nebaigtas vidurinis

○ Vidurinis

○ Profesinis

○ Aukštasis

5. **Šeimyninė padėtis**

○ Gyvenu viena/ vienas

○ Gyvenu viena/ vienas ir auginu vaiką (-us)

○ Gyvenu su sutuoktine (-iu) / partnere (-iu), vaikų neauginame

○ Gyvenu su sutuoktine (-iu) / partnere (-iu) ir kartu auginame vaiką (-us)

○ Kita (*įrašykite*):

6. **Gyvenamoji vieta**

○ Sostinė/ rajono centras

○ Kita

7. **Ar šiuo metu vartojate kokius nors nikotino turinčius produktus (pvz., cigaretės, elektroninės cigaretės, nikotino pleistras/guma ir pan.)?**

○ Taip

○ Ne

8. **Ar Jūs, Jūsų asmenine nuomone, esate pakankamai fiziškai aktyvus?**

○ Taip

○ Ne

9. **Ar Jūs, Jūsų asmenine nuomone, sveikai maitinatės?**

○ Taip

○ Ne

10. Ar be migrenos turite kitų sveikatos sutrikimų (miego, nuotaikos) ar sergate kita kokia nors liga (ligomis)? *Pasirinkite visus tinkamus atsakymus*

☐ Ne

☐ Kiti neurologiniai susirgimai (epilepsija, išsėtinė sklerozė, insultas ir kt.)

☐ Miego sutrikimai (nemiga, miego apnėja, neramių kojų sindromas ir kt.)

☐ Nerimas

☐ Depresija

☐ Endokrininiai susirgimai (skydliaukės funkcijos sutrikimai, cukrinis diabetas ir kt.)

☐ Širdies kraujagyslių ligos ar padidėjusi jų rizika (arterinė hipertenzija (padidėjęs kraujo spaudimas), cholesterolio padidėjimas, dažnas širdies plakimas ir kt.)

☐ Virškinamojo trakto ligos (refliuksas, gastritas, opaligė, dirgliosios žarnos sindromas, hemorojus ir kt.)

☐ Alerginės ligos (bronchinė astma, šienligė, odos alerginės ligos ir kt.)

☐ Sąnarių ir sisteminės jungiamojo audinio ligos (artrozė, reaktyvusis artritas, psoriatinė artropatija, reumatoidinis artritas, sisteminė raudonoji vilkligė ir kt.)

☐ Odos ligos (psoriazė, egzema ir kt.)

☐ Ginekologiniai susirgimai (endometriozė, policistinių kiaušidžių sindromas, mioma ir kt.)

☐ Onkologiniai susirgimai

☐ Kita (*įrašykite*):

11. Ar galite apskritai sakyti, kad Jūsų sveikata yra:

☐ Puiki

☐ Labai gera

☐ Gera

☐ Nebloga

☐ Bloga

2. INFORMACIJA APIE GALVOS SKAUSMĄ IR JO TRADICINĮ MEDIKAMENTINĮ GYDYMĄ

12. Ar turite nustatytą migrenos diagnozę?

○ Taip

○ Ne

13. Kokio amžiaus Jums prasidėjo migrenos priepuoliai?

____________ m.

14. Kokia migrena Jums diagnozuota?
Pažymėkite visus tinkamus

☐ Migrena su aura

☐ Migrena be auros

☐ Lėtinė migrena

☐ Vestibulinė migrena

☐ Menstruacinė migrena

☐ Nei viena iš paminėtų

☐ Migrena man yra diagnozuota, bet nežinau kokia

15. Ar Jums kada nors buvo diagnozuotas ilgai vartojamų vaistų sukeltas galvos skausmas?

○ Taip

○ Ne

○ Nežinau

16. Kaip dažnai patiriate galvos skausmą (bet kokio stiprumo galvos skausmas)?

○ Rečiau nei 1 dieną per mėnesį

○ 1 dieną per mėnesį, rečiau nei 1 dieną per savaitę

○ Vidutiniškai 1 dieną per savaitę

○ 2-3 dienas per savaitę

○ 4-5 dienas per savaitę

○ Beveik kiekvieną dieną/ kiekvieną dieną

17. Įvertinkite nuo 1 iki 10 vidutinį galvos skausmo, kurio nepavyko numalšinti vaistais, stiprumą (1 – minimalus, 10 – stipriausias įsivaizduojamas):

18. Vidutinė galvos skausmo trukmė?

○ Iki 1 val.

○ 1-4 val.

○ 4-12 val.

○ 12-24 val.

○ Ilgiau nei 24 val.

19. Ar vartojate vaistus nuo skausmo migrenos priepuoliui malšinti?

○ Taip

○ Ne

20. Kaip PER PASTARUOSIUS 12 MĖN. gydote migrenos priepuolius?
Pažymėkite visus tinkamus atsakymus

☐ Geriate aspiriną

☐ Geriate paracetamolį

☐ Geriate kitus nesteroidinius vaistus nuo uždegimo (pvz., ibuprofenas, diklofenakakas, naproksenas, ir kt.)

☐ Geriate sudėtinius analgetikus su kofeinu ar kodeinu (pvz., Citramon, Saridon, Askofen, Solpadein)

☐ Geriate triptanus (pvz., sumatriptanum (Imigran, Cinie), frovatriptanum (Migard), rizatriptanum (Maxalt), zolmitriptanum (Zomig), naratriptanum (Naramig, Naratriptan), almotriptanum)

☐Leidžiate vaistus į raumenis (pvz. diklofenacum (Naklofen), ketorolakum (Ketanov), dexketoprofenum (Dolmen), metamizolum (Analgin), metoclopramidum (Cerucal), dexamethasonum)

☐Geriate raminamuosius vaistus (pvz. bromazepamum (Lexotanil), alprazolamum (Xanax), diazepamum (Relanium) it kt.)

☐Kviečiate greitąją medicinos pagalbą

☐Pats/pati vykstate į priėmimo skubios pagalbos skyrių

☐ Kita (*įrašykite*):

21. Ar Jūsų migrenos priepuolio gydymas veiksmingas? (vaistai veiksmingi, jei skausmas praeina ar reikšmingai sumažėja per 2 val.)

- ○ Taip, visada
- ○ Taip, dažniausiai
- ○ Dažniausiai neveiksmingi
- ○ Neveiksmingi

22. Ar gydytojo paskirtus vaistus migrenos PRIEPUOLIUI GYDYTI vartojate TIKSLIAI, kaip buvo nurodęs gydytojas?

- ○ Visada pagal gydytojo rekomendacijas
- ○ Dažniausiai pagal gydytojo rekomendacijas
- ○ Kartais pagal gydytojo rekomendacijas, dažniau vartoju savo nuožiūra
- ○ Labai retai pagal gydytojo rekomendacijas, dažniausiai vartoju savo nuožiūra
- ○ Vaistus vartoju savo nuožiūra

23. Ar ŠIUO METU vartojate migreną retinančių vaistų (tai yra vadinama profilaktika)? Jei taip, tai kokius? *Galite pasirinkti daugiau nei vieną atsakymą.*

☐Ne

☐Beta blokatorius: metoprololį (Betaloc, Corvitol, Emzok)/ propranololį

☐Vaistus nuo epilepsijos: valproine rūgštį (Depakine chrono)/ topiramatą (Topamax)

☐Antidepresantus: amitriptiliną (*Amytriptilin*)/ venlafaksiną (*Alventa, Lanvexin, EfexorI)*/ nortriptiliną (*Noritren*)

☐AKF veikiančius vaistus: kandesartaną (Candesartan, Canocombi, Canocord)/ lizinoprilį (Dironorm, Diroton)

☐Botulinas (Botox), injekcijas atliko gydytojas neurologas (31-39 dūriai vienos procedūros metu)

☐Botulinas (Botox, Dysport), injekcijas atliko grožio specialistas ar gydytojas ne neurologas (keli dūriai kaktos srityje)

☐ Erenumabas (Aimovig), Fremanezumabas (Ajovy), Galkanezumabas (Emgality)

24. Ar gydytojo skirtus vaistus migrenai retinti, t.y. PROFILAKTIKAI vartojate TIKSLIAI, kaip buvo nurodęs gydytojas?

- ○ Visada pagal gydytojo rekomendacijas
- ○ Dažniausiai pagal gydytojo rekomendacijas
- ○ Kartais pagal gydytojo rekomendacijas, dažniau vartoju savo nuožiūra
- ○ Labai retai pagal gydytojo rekomendacijas, dažniausiai vartoju savo nuožiūra
- ○ Vaistus vartoju savo nuožiūra
- ○ Vaistų nevartoju

25. Ar migrena turi poveikį Jūsų darbingumui?

- ○ Taip, visada esu nedarbinga (-s)
- ○ Taip, dažniausiai esu nedarbinga (-s)
- ○ Retai esu nedarbinga (-s)
- ○ Poveikio darbingumui nėra

26. Ar dėl galvos skausmo yra tekę imti nedarbingumo pažymėjimą?

- ○ Taip, visada
- ○ Taip, dažniausiai
- ○ Taip, retai
- ○ Ne

3. ALTERNATYVIOS MEDICINOS PRIEMONIŲ (AMP) NAUDOJIMAS

Alternatyvios medicinos priemonės - visos neprofesinės, t.y. mokslinės medicinos neįteisintos, gydymo priemonės ir metodai, mėginantys pakeisti ar papildyti profesionaliąją mediciną. Šios apklausos metu alternatyvios medicinos priemonėmis laikykime visus įmanomus veiksmus, medžiagas ar įrenginius, kuriuos vartojant/ naudojant tikimasi sumažinti migrenos galvos skausmą ar sumažinti priepuolių dažnį, išskyrus gydytojo paskirtus vaistus ir prietaisus. **Šioms priemonėms priskiriami fiziniai pratimai, akupunktūra, masažai, homeopatija, žoliniai preparatai ir medžiagos, atsipalaidavimo būdai, vitaminai, mineralai, įvairios dietos, badavimas ir panašiai.**

27. Ar sutinkate su teiginiu, kad Jūsų pasveikimas labiausiai priklauso nuo Jūsų pačio (-s), o ne medikų veiksmų?

- ○ Visiškai sutinku
- ○ Labiau sutinku, nei nesutinku
- ○ Nežinau/ Negaliu atsakyti
- ○ Labiau nesutinku, nei sutinku
- ○ Visiškai nesutinku

28. Ar sutinkate su teiginiu, kad alternatyvios medicinos priemonės yra natūralios?

- ○ Visiškai sutinku
- ○ Labiau sutinku, nei nesutinku
- ○ Nežinau/ Negaliu atsakyti
- ○ Labiau nesutinku, nei sutinku
- ○ Visiškai nesutinku

29. Ar sutinkate su teiginiu, kad alternatyvios medicinos priemonės yra saugesnės nei gydytojų skiriami vaistai?

- ○ Visiškai sutinku
- ○ Labiau sutinku, nei nesutinku
- ○ Nežinau/ Negaliu atsakyti
- ○ Labiau nesutinku, nei sutinku
- ○ Visiškai nesutinku

30. Ar esate KADA NORS naudojusi(-ęs) alternatyvios medicinos priemones migrenos gydymui ir/ar prevencijai?

- ○ Taip, migrenos skausmui gydyti
- ○ Taip, migrenos priepuoliams retinti
- ○ Taip, migrenos skausmui gydyti ir priepuoliams retinti
- ○ Ne

31. Dėl kokių priežasčių nusprendėte išbandyti alternatyvias medicinos priemones migrenos skausmo gydymui/ profilaktikai?
Galite pasirinkti daugiau nei vieną atsakymą.

☐ Gydytojo paskirti vaistai buvo mažai veiksmingi

☐ Gydytojo paskirti vaistai buvo visiškai neveiksmingi

☐ Norėjau rečiau vartoti gydytojo paskirtus vaistus, nes jie turi nepageidaujamą poveikį

☐ Norėjau rečiau vartoti gydytojo paskirtus vaistus, nes, manau, kad jie gali turėti neigiamos įtakos mano sveikatai

☐ Norėjau rečiau vartoti gydytojo paskirtus vaistus, nes jie yra brangūs

☐ Vartojau norėdama (-s) sumažinti nepageidaujamą gydytojo paskirtų vaistų poveikį

☐ Vartojau , nes nebuvo galimybės nusipirkti gydytojų skiriamų vaistų

☐ Kita(*įrašykite*):

32. Ar per pastaruosius 12 MĖN. migrenos skausmui gydyti ir/ar priepuoliams retinti (profilaktikai) vartojote alternatyvios medicinos priemonių iš grupės: GERIAMIEJI PREPARATAI (pvz., fitoterapija, vaistažolės, arbatos, homeopatiniai preparatai, natūralios medžiagos, tradicinės rytų medicinos preparatai ir panašiai, išskyrus kanapių geriamieji preparatai)?

- ○ Taip
- ○ Ne

33. Gal galėtumėte įvardinti kokius geriamuosius preparatus vartojote migrenos skausmui gydyti/ priepuoliams retinti?

34. Kokiu tikslu vartojote šių alternatyvios medicinos priemonių?

- ○ Migrenos skausmui gydyti

○ Migrenos skausmui retinti (profilaktikai)

○ Dėl abiejų priežasčių

35. Ar per pastaruosius 12 MĖN. migrenos skausmui gydyti ir/ar priepuoliams retinti (profilaktikai) vartojote alternatyvios medicinos priemonių iš grupės: KANAPIŲ RŪKYMAS/ KANAPIŲ PREPARATAI?

○ Taip

○ Ne

36. Galbūt galėtumėte įvardinti, kokius kanapių preparatus naudojote?
Galite pasirinkti daugiau nei vieną atsakymą.

☐ Kanapių rūkymas

☐ Kanabidiolio (CBD) aliejaus lašiukai į burną

☐ Maisto papildai iš kanapių sėklų

☐ Kita (*įrašykite*):

37. Kokiu tikslu vartojote šių alternatyvios medicinos priemonių?

○ Migrenos skausmui gydyti

○ Migrenos skausmui retinti (profilaktikai)

○ Dėl abiejų priežasčių

38. Ar per pastaruosius 12 MĖN. migrenos skausmui gydyti ir/ar priepuoliams retinti (profilaktikai) vartojote alternatyvios medicinos priemonių iš grupės: ANT KŪNO DEDAMI/ TEPAMI PREPARATAI (pvz., įvairūs eteriniai aliejai, tepalai, kremai, kompresai ir panašiai)?

○ Taip

○ Ne

39. Gal galėtumėte įvardinti kokius ant kūno dedamus/ tepamus preparatus naudojote?
Galite pasirinkti daugiau nei vieną atsakymą

☐ Šaldančios aplikacijos (ledas, šalta veido kaukė ir pan.)

☐ Šildančios aplikacijos (pvz., karšto vandens pūslė, įvairios šildyklės, šildančios pagalvės ir pan.)

☐ Eteriniai aliejai (pvz., levandų, eukalipto, pipirmėtės ir pan.)

☐ Šildantys, šaldantys kremai

☐ Kitokio poveikio kremai

☐ Įvairūs kompresai

☐ Kita (*įrašykite*):

40. Kokiu tikslu vartojote šių alternatyvios medicinos priemonių?

○ Migrenos skausmui gydyti

○ Migrenos skausmui retinti (profilaktikai)

○ Dėl abiejų priežasčių

41. Ar per pastaruosius 12 MĖN. migrenos skausmui gydyti ir/ar priepuoliams retinti (profilaktikai) laikėtės SPECIALIOS DIETOS/ BADAVOTE/ ATSISAKĖTE TAM TIKRŲ PRODUKTŲ MAISTO PRODUKTŲ?

- ○ Taip
- ○ Ne

42. Galbūt galėtumėte dietą įvardinti?
Galite pasirinkti daugiau nei vieną atsakymą

- ☐ Badavau
- ☐ Viduržemio jūros dieta
- ☐ Ketogeninė dieta
- ☐ Mažai angliavandenių/ cukraus turinti dieta
- ☐ Dieta be laktozės
- ☐ Veganiška mityba
- ☐ Vegetariška mityba
- ☐ DASH dieta
- ☐ Kita (*įrašykite*):

43. Kokiu tikslu laikėtės dietos/ badavote?

- ○ Migrenos skausmui gydyti
- ○ Migrenos skausmui retinti (profilaktikai)
- ○ Dėl abiejų priežasčių

44. Ar per pastaruosius 12 MĖN. migrenos skausmui gydyti ir/ar priepuoliams retinti (profilaktikai) naudojote kokį nors ATSIPALAIDAVIMO BŪDĄ (pvz., meditacija, aromaterapija, meno, spalvų, muzikos terapija, vizualizacija ir pan.)?

- ○ Taip
- ○ Ne

45. Kokius ATSIPALAIDAVIMO BŪDUS PER PASTARUOSIUS 12 MĖN. naudojote migrenos skausmui gydyti/ priepuoliams retinti?
Galite pasirinkti daugiau nei vieną atsakymą

- ☐ Vandens procedūros
- ☐ Aromaterapija (kvapų terapija)
- ☐ Meditacija
- ☐ Muzikos terapija
- ☐ Meno terapija
- ☐ Spalvų terapija
- ☐ Vizualizacija
- ☐ Atsipalaidavimo/ kvėpavimo pratimai

☐ Kita (*įrašykite*):

46. Kokiu tikslu naudojote šią alternatyvios medicinos priemonę?

- O Migrenos skausmui gydyti
- O Migrenos skausmui retinti (profilaktikai)
- O Dėl abiejų priežasčių

47. Ar per pastaruosius 12 MĖN. migrenos skausmui gydyti ir/ar priepuoliams retinti naudojote kokį nors FIZINĮ AKTYVUMĄ (pvz., joga, tempimo pratimai, šokiai, bėgimas, pasivaikščiojimas ir pan.)?

- O Taip
- O Ne

48. Kokį FIZINĮ AKTYVUMĄ PER PASTARUOSIUS 12 MĖNESIŲ pasirinkote migrenos skausmui gydyti/ priepuoliams retinti?
Galite pasirinkti daugiau nei vieną atsakymą

☐ Šokio ir/ ar judesių terapija

☐ Joga/ tempimo pratimai

☐ Tai chi

☐ Plaukimas

☐ Bėgiojimas

☐ Aerobiniai pratimai

☐ Ėjimas pasivaikščioti

☐ Kita (*įrašykite*):

49. Kokiu tikslu naudojote šiuos fizinius pratimus?

- O Migrenos skausmui gydyti
- O Migrenos skausmui retinti (profilaktikai)
- O Dėl abiejų priežasčių

50. Ar per pastaruosius 12 MĖN. migrenos skausmui gydyti ir/ar priepuoliams retinti Jums buvo atlikta AKUPUNKTŪRA (adatomis, elektroakupunktūra, termopunktūra, aplikacinė refleksoterapija ir pan.) ir/ar AKUPRESŪRA?

- O Taip
- O Ne

51. Kokiu tikslu naudojote šią alternatyvios medicinos priemonę?

- O Migrenos skausmui gydyti
- O Migrenos skausmui retinti (profilaktikai)
- O Dėl abiejų priežasčių

52. Ar per pastaruosius 12 MĖN. migrenos skausmui gydyti ir/ar priepuoliams retinti naudojote MASAŽĄ ir/ar CHIROPRAKTIKĄ ir/ar OSTEOPATIJĄ?

- O Taip

○ Ne

53. Kokias alternatyvios medicinos priemones naudojote?
Galite pasirinkti daugiau nei vieną atsakymą

☐ Masažą

☐ Chiropraktiką

☐ Osteopatiją

54. Kokiu tikslu naudojote šią alternatyvios medicinos priemonę?

○ Migrenos skausmui gydyti

○ Migrenos skausmui retinti (profilaktikai)

○ Dėl abiejų priežasčių

55. Ar informavote savo gydytoją, kad migrenos priepuoliams gydyti ir/ar jiems retinti vartojate/naudojate alternatyvios medicinos priemones?

○ Taip

○ Ne

Šiuolaikinių sveikatos rūpesčių (Modern Health Worries) klausimynas
Prašytume Jūsų užpildyti „Šiuolaikinių sveikatos rūpesčių“ klausimyną.

56. Ar Jums kelia susirūpinimą tai, jog įvairiomis medžiagomis (pesticidais, fluoridais, nitratais ir pan.) užterštas vanduo gali kenkti Jūsų sveikatai?

○ Kelia didelį susirūpinimą/ beveik nuolat apie tai galvoju

○ Kelia vidutinio dydžio susirūpinimą dažnai apie tai galvoju

○ Nežinau/ Negaliu atsakyti

○ Kelia mažą susirūpinimą/ retai apie tai galvoju

○ Susirūpinimo nekelia

57. Ar Jums kelia susirūpinimą tai, jog elektroninių prietaisų (mikrobangų krosnelių, kompiuterių, telefonų, radijo bangų stočių, ryšio bokštų ir pan.) skleidžiama spinduliuotė gali kenkti Jūsų sveikatai?

○ Kelia didelį susirūpinimą/ beveik nuolat apie tai galvoju

○ Kelia vidutinio dydžio susirūpinimą dažnai apie tai galvoju

○ Nežinau/ Negaliu atsakyti

○ Kelia mažą susirūpinimą/ retai apie tai galvoju

○ Susirūpinimo nekelia

58. Ar Jums kelia susirūpinimą tai, jog medicinos diagnostinių tyrimų metu naudojamų prietaisų (rentgeno, kompiuterinės tomografijos ir pan.) skleidžiama radiacija gali kenkti Jūsų sveikatai?

○ Kelia didelį susirūpinimą/ beveik nuolat apie tai galvoju

○ Kelia vidutinio dydžio susirūpinimą dažnai apie tai galvoju

○ Nežinau/ Negaliu atsakyti

○ Kelia mažą susirūpinimą/ retai apie tai galvoju

○ Susirūpinimo nekelia

59. Ar Jums kelia susirūpinimą tai, jog maiste esančios medžiagos (pvz., mikroplastikas, bakterijos, įvairiais priedai, pesticidai, antibiotikai) ir/ar genetiškai modifikuoti produktai gali kenkti Jūsų sveikatai?

- O Kelia didelį susirūpinimą/ beveik nuolat apie tai galvoju
- O Kelia vidutinio dydžio susirūpinimą dažnai apie tai galvoju
- O Nežinau/ Negaliu atsakyti
- O Kelia mažą susirūpinimą/ retai apie tai galvoju
- O Susirūpinimo nekelia

60. Ar Jums kelia susirūpinimą tai, jog įvairios vakcinos gali kenkti Jūsų sveikatai?

- O Kelia didelį susirūpinimą/ beveik nuolat apie tai galvoju
- O Kelia vidutinio dydžio susirūpinimą dažnai apie tai galvoju
- O Nežinau/ Negaliu atsakyti
- O Kelia mažą susirūpinimą/ retai apie tai galvoju
- O Susirūpinimo nekelia

Didžiojo penketo asmenybės dimensijos (DPAD)

Prašytume Jūsų užpildyti „Didžiojo penketo asmenybės dimensijų“ klausimyną.

Kiekvienai būdvardžių porai apibraukite skaitmenį skalėje, kuris tiksliausiai atspindi jūsų asmenybę. Pavyzdžiui: sąžiningas 1 2 3 4 5 6 7 nesąžiningas. Jeigu laikote save labai sąžiningu, apibraukite 1 arba 2, jeigu laikote save labai nesąžiningu, apibraukite 6 arba 7. Jeigu Jūsų sąžiningumas priklauso nuo aplinkybių, apibraukite 3, 4 arba 5, priklauso nuo to, kas jums labiau tinka.

(A)Mėgstantis bendrauti	1	2	3	4	5	6	7	Mėgstantis vienumą
(B)Ramus	1	2	3	4	5	6	7	Susirūpinęs
(C) Originalus	1	2	3	4	5	6	7	Įprastas
(D) Sąžiningas	1	2	3	4	5	6	7	Aplaidus
(E) Dirglus	1	2	3	4	5	6	7	Gero būdo
(F) Rimtas	1	2	3	4	5	6	7	Mėgstantis juokus
(G) Nervingas	1	2	3	4	5	6	7	Pakantus
(H) Tikroviškas	1	2	3	4	5	6	7	Lakios vaizduotės
(I) Nerūpestingas	1	2	3	4	5	6	7	Atsargus
(J) Minkštaširdis	1	2	3	4	5	6	7	Kietaširdis
(K) Meilus	1	2	3	4	5	6	7	Santūrus
(L) Atsipalaidavęs	1	2	3	4	5	6	7	Įsitempęs
(M) Kūrybingas	1	2	3	4	5	6	7	Nekūrybingas
(N) Patikimas	1	2	3	4	5	6	7	Nepatikimas
(O) Šiurkštus	1	2	3	4	5	6	7	Paslaugus
(P) Abejingas	1	2	3	4	5	6	7	Draugiškas
(Q) Saugus	1	2	3	4	5	6	7	Nesaugus
(R) Siaurų interesų	1	2	3	4	5	6	7	Plačių interesų
(S) Neorganizuotas	1	2	3	4	5	6	7	Organizuotas
(T) Atlaidus	1	2	3	4	5	6	7	Kerštingas
(U) Spontaniškas	1	2	3	4	5	6	7	Suvaržytas
(V) Savigaila	1	2	3	4	5	6	7	Patenkintas savimi
(W) Sudėtingas	1	2	3	4	5	6	7	Paprastas
(X) Valingas	1	2	3	4	5	6	7	Silpnavalis
(Y) Bejausmis	1	2	3	4	5	6	7	Nuoširdus
